# Selenium Attenuates LPS-Induced Injury in Ovine Granulosa Cells by Protecting Mitochondrial Ultrastructure and Cellular Homeostasis

**DOI:** 10.3390/ani16132095

**Published:** 2026-07-06

**Authors:** Zeyuan Guo, Jun Li, Xinyu Fan, Yufei Liu, Linzhen Li, Lihua Lyu, Chunhe Yang, Youshen Ren

**Affiliations:** College of Animal Science, Shanxi Agricultural University, Taigu, Jinzhong 030801, China; gzy15581741973@163.com (Z.G.); 15182767265@163.com (J.L.); 18835454822@139.com (X.F.); 18335416734@163.com (Y.L.); 15534785520@163.com (L.L.); lihualvsxau@126.com (L.L.); ych2019@sxau.edu.cn (C.Y.)

**Keywords:** ovine, GCs, selenium, mitochondrial ultrastructure, inflammation, oxidative stress, apoptosis, steroidogenesis

## Abstract

Lipopolysaccharide (LPS) impairs the function of ovine follicular granulosa cells (GCs), representing a primary cause of follicular atresia. Selenium (Se), an essential trace element, possesses anti-inflammatory and cytoprotective properties; however, its effects on GC ultrastructure remain largely unknown. In this study, primary ovine GCs were exposed to LPS (10 µg/mL) and treated with sodium selenite (25 nM). Transmission electron microscopy (TEM), JC-1 staining, enzyme-linked immunosorbent assay (ELISA), reactive oxygen species (ROS) detection, flow cytometry, and quantitative real-time PCR (qRT-PCR) were employed to evaluate cellular ultrastructure, mitochondrial membrane potential (ΔΨm), and downstream physiological processes. LPS induced severe mitochondrial pyknosis, cristae loss, and reduced ΔΨm, accompanied by inflammation, oxidative stress, apoptosis, and impaired steroidogenesis. Se intervention markedly ameliorated these ultrastructural injuries, preserving mitochondrial morphology and ΔΨm. Functionally, Se suppressed the release of tumor necrosis factor-alpha (TNF-α), interleukin-6 (IL-6), and interleukin-1 beta (IL-1β); enhanced the activities of antioxidant enzymes including superoxide dismutase (SOD), glutathione peroxidase (GSH-Px), and catalase (CAT) while attenuating ROS accumulation; inhibited apoptosis by upregulating *BCL-2* and downregulating *BAX* and *CASPASE-3*; and restored E_2_ and P_4_ secretion via upregulation of *STAR* and *NR5A1*. This study provides direct morphological evidence that Se protects ovine GCs from LPS-induced damage by repairing mitochondrial ultrastructure. This structural restoration is central to its integrated anti-inflammatory, antioxidant, anti-apoptotic, and steroidogenic effects. These in vitro findings suggest that Se may serve as a promising nutritional strategy for mitigating inflammation-driven follicular atresia, pending further in vivo validation.

## Simple Summary

In sheep farming, ovarian inflammation caused by bacterial infections often leads to infertility and economic losses. Bacterial toxins damage the ovarian cells that support egg development, but whether selenium, a trace mineral with recognized anti-inflammatory properties, can protect these cells remains unclear. In this study, we exposed sheep ovarian cells to bacterial toxin to induce inflammation and then treated them with selenium. Using high-resolution microscopy and functional assays, we found that the toxin severely damaged the mitochondria—the energy-producing compartments of the cells—causing them to shrink and fragment. Selenium treatment restored these structures to a healthy state. This structural repair, in turn, reduced inflammation and cell death, alleviated oxidative stress, and improved the production of reproductive hormones by the cells. Our findings provide direct evidence that selenium protects sheep ovarian cells by preserving their internal structures, suggesting that selenium supplementation could serve as a practical nutritional strategy for safeguarding sheep fertility against inflammation-related disorders.

## 1. Introduction

Ovarian follicular granulosa cells (GCs) constitute the largest cell population within the ovary and serve as the primary source of steroid hormones [1]. As the somatic cells most intimately associated with oocytes, GCs provide nutritional support for oocyte growth and maturation, mediate intercellular communication within the follicular microenvironment, and maintain a stable milieu essential for folliculogenesis and ovulation [2,3]. Being high-energy-demand cells, GCs rely heavily on mitochondrial functional integrity to sustain proliferation, differentiation, and steroidogenesis [4]. Accumulating evidence suggests that pro-inflammatory insults within the follicular microenvironment disrupt mitochondrial homeostasis, leading to GC dysfunction [5,6]. This inflammatory cascade further exacerbates mitochondrial damage, creating a vicious cycle that ultimately drives follicular atresia [7].

Lipopolysaccharide (LPS), a major endotoxin derived from Gram-negative bacteria, is a well-established inducer of ovarian inflammation [8]. In intensive sheep production systems, LPS activates the Toll-like receptor 4 (TLR4) pathway in bovine GCs, inhibiting progesterone secretion and impairing oocyte meiotic progression [9,10], and promotes the release of pro-inflammatory cytokines that further exacerbate GC dysfunction [11]. This LPS-driven inflammatory cascade is closely associated with reduced fertility in sheep flocks and leads to substantial economic losses in the livestock industry.

At the subcellular level, mitochondrial dysfunction is increasingly recognized as a central mediator of LPS-induced cellular injury [12]. Mitochondrial quality control is maintained through two coordinated mechanisms: the selective degradation of damaged mitochondria via mitophagy, primarily governed by the PARKIN/PINK1 pathway [13], and the dynamic balance between mitochondrial fission and fusion, which is regulated by key proteins such as dynamin-related protein 1 (DRP1), mitofusin-1/mitofusin-2 (MFN1/MFN2), and optic atrophy protein 1 (OPA1) [14]. Disruption of either process leads to the accumulation of dysfunctional mitochondria, excessive ROS production, and ultimately the activation of apoptotic pathways [15]. Whether Se can restore mitochondrial quality control by modulating these pathways in LPS-challenged ovine GCs has not been investigated.

Selenium (Se) is an essential trace element that plays critical roles in antioxidant defense, anti-inflammatory responses, and reproductive regulation, largely mediated through its incorporation into selenoproteins [16,17]. In livestock, dietary Se supplementation improves reproductive performance, antioxidant capacity, and immune function [18,19,20], whereas Se deficiency increases the incidence of reproductive disorders [21]. At the cellular level, Se stimulates estradiol biosynthesis in ovine GCs [22] and attenuates LPS-induced inflammatory injury in bovine endometrial epithelial cells via modulation of TLR4, phosphoinositide 3-kinase (PI3K)/protein kinase B (AKT), and wingless-related integration site (Wnt)/β-catenin pathways [23,24]. However, whether Se can protect against LPS-induced ovarian GC injury by preserving mitochondrial ultrastructure and restoring mitophagy and mitochondrial dynamics remains unknown.

Therefore, the present study aimed to investigate the protective effects of Se on mitochondrial membrane potential (ΔΨm), mitophagy-related markers, and dynamics-associated proteins in an LPS-induced ovine GC inflammation model, and to elucidate the potential mechanisms linking mitochondrial quality control to the preservation of cellular function.

## 2. Materials and Methods

### 2.1. Isolation, Culture, and Treatment of Primary Ovine GCs

Ovine ovaries were collected aseptically and transported to the laboratory in sterile saline containing 1% antibiotics. Follicular fluid from healthy follicles (3–6 mm) was aspirated and centrifuged (1000 rpm, 5 min), and the cell pellet was washed twice with PBS. Cells were resuspended in DMEM/F12 supplemented with 10% FBS (CellMax, Beijing, China) and 1% antibiotics, and cultured at 37 °C in 5% CO_2_. Granulosa cell purity was verified by follicle-stimulating hormone receptor (FSHR) immunofluorescence. GCs in the logarithmic growth phase (1 × 10^6^ cells/mL) were divided into three groups: Control (complete medium), LPS (10 μg/mL LPS for 12 h followed by complete medium for 24 h), and LPS + Se (10 μg/mL LPS for 12 h followed by 25 nM sodium selenite for 24 h). Sodium selenite was used as the selenium source. All reported concentrations refer to the final concentration of sodium selenite in the culture medium. LPS and sodium selenite were obtained from Sigma-Aldrich (St. Louis, MO, USA).

### 2.2. Immunofluorescence Staining

GCs were seeded onto coverslips placed in 6-well plates and incubated overnight at 37 °C with 5% CO_2_. Cells were fixed with 4% paraformaldehyde (Biosharp, Wuhan, China), permeabilized with 0.3% Triton X-100, and blocked with 5% BSA. Subsequently, cells were incubated with anti-FSHR primary antibody (1:50) overnight at 4 °C, followed by FITC-conjugated secondary antibody (1:200) for 1 h in the dark. Nuclei were counterstained with DAPI. FSHR expression was visualized using a fluorescence microscope.

### 2.3. Cell Counting Kit-8 (CCK-8) Assay

GCs in the logarithmic growth phase were seeded into 96-well plates at a density of 1 × 10^4^ cells per well and cultured for 24 h. To determine the optimal concentrations of LPS and sodium selenite, cells were treated with different concentration gradients of sodium selenite (0, 25, 50, 100, 200, 500, and 1000 nM) for 24 or 48 h, or with LPS (0, 0.01, 0.1, 1, 10, and 100 μg/mL) for 12 or 24 h. Subsequently, 10 μL of CCK-8 reagent (Meilunbio, Shanghai, China) was added to each well and incubated at 37 °C for 3–4 h. The absorbance was measured at 450 nm using a microplate reader. Three independent experiments were performed.

### 2.4. Enzyme-Linked Immunosorbent Assay (ELISA)

Concentrations of tumor necrosis factor-alpha (TNF-α), interleukin-1 beta (IL-1β), interleukin-6 (IL-6), estradiol (E_2_), and progesterone (P_4_) in culture supernatants were quantified using commercial ELISA kits (Enzymelink, Shanghai, China). Diluted standards or samples were added to wells, followed by biotin-conjugated detection antibody. After incubation at 37 °C for 1 h, wells were washed, and streptavidin-HRP conjugate was added for 30 min at 37 °C. Substrates A and B were then added and incubated for 10 min in the dark. The reaction was terminated with stop solution, and absorbance was measured at 450 nm. Sample concentrations were calculated from standard curves using four-parameter logistic curve fitting.

### 2.5. Mitochondrial Membrane Potential (ΔΨm) Detection

ΔΨm was assessed using the JC-1 fluorescent probe (Beyotime, Shanghai, China). GCs in the logarithmic growth phase were seeded into 6-well plates. Based on CCK-8 dose–response assays, 10 μg/mL LPS (12 h) was selected as the optimal inflammatory challenge concentration, as it robustly induced pro-inflammatory cytokine expression without significantly compromising cell viability, and 25 nM sodium selenite was selected as the optimal protective concentration, as it maximally restored the viability of LPS-injured granulosa cells. Cells were treated with 10 μg/mL LPS for 12 h, followed by 25 nM sodium selenite for 24 h. After treatment, cells were incubated with JC-1 staining working solution at 37 °C for 20 min, washed twice with JC-1 staining buffer, and immediately imaged under a laser confocal microscope.

### 2.6. Reactive Oxygen Species (ROS) Detection

Intracellular ROS levels were measured using the DCFH-DA probe (Beyotime, Shanghai, China). GCs in the logarithmic growth phase were seeded into 96-well plates and treated with 10 μg/mL LPS for 12 h, followed by 25 nM sodium selenite for 24 h. The culture medium was replaced with serum-free medium containing 10 μM DCFH-DA, and cells were incubated at 37 °C in the dark for 20 min. After three washes with PBS, green fluorescence was observed under a fluorescence microscope, and fluorescence intensity was quantified using a microplate reader (excitation 488 nm, emission 525 nm).

### 2.7. Annexin V-FITC/PI/Hoechst 33342 Apoptosis Assay

GCs in the logarithmic growth phase were seeded in 6-well plates, treated with 10 μg/mL LPS for 12 h, and then with 25 nM sodium selenite for 24 h. Cells were harvested with EDTA-free trypsin, washed twice with ice-cold PBS, and resuspended in 1× Binding Buffer at a density of 1–5 × 10^5^ cells/mL. The cells were stained with Annexin V-FITC, PI (Yeasen, Shanghai, China), and Hoechst 33342 according to the manufacturer’s instructions, incubated at room temperature in the dark for 15 min, and then diluted with 400 μL ice-cold 1× Binding Buffer. Fluorescence was examined immediately using a fluorescence microscope. For each sample, six non-overlapping fields were randomly selected and photographed. Total cells (Hoechst 33342-positive), early apoptotic cells (Annexin V-FITC-positive, PI-negative), and late apoptotic or necrotic cells (Annexin V-FITC-positive and PI-positive) were counted using the ImageJ 1.47V. The apoptotic rates were calculated as the number of early or late apoptotic cells divided by the total cell number × 100%. All image analyses were performed in a blinded manner.

### 2.8. Transmission Electron Microscopy (TEM)

Cells were pelleted, fixed with electron microscopy fixative at 4 °C for 2–4 h, and washed three times with 0.1 M phosphate buffer (PB, pH 7.4). The pellet was embedded in 1% agarose, post-fixed with 1% osmium acid in 0.1 M PB in the dark at room temperature for 2 h, rinsed with PB, dehydrated through a graded ethanol series (30–100%) and 100% acetone, infiltrated with a mixture of acetone and 812 embedding agent (1:1 for 2–4 h, 1:2 overnight, and pure 812 for 5–8 h at 37 °C), and polymerized in pure 812 at 60 °C for 48 h. Ultrathin sections (60–80 nm) were cut with an ultramicrotome, collected on 150-mesh copper grids, stained with 2% uranyl acetate-ethanol for 8 min in the dark and with 2.6% lead citrate for 8 min, washed, dried, and observed under a transmission electron microscope(HITACHI, Tokyo, Japan).

For quantitative analysis of mitochondrial morphology, ovarian GCs were isolated from 20 healthy female sheep (4–6 months of age) with similar body condition scores and normal estrous cycles. After LPS and Se treatments, five cell profiles were analyzed per treatment group, and five mitochondria per group were manually outlined along the outer membrane using ImageJ software to measure mitochondrial area and aspect ratio (major axis/minor axis). All measurements were performed in a blinded manner, with the observer unaware of the experimental group allocation during image analysis.

### 2.9. Catalase (CAT) Activity Detection

A 5 mM hydrogen peroxide (H_2_O_2_) solution and a chromogenic working solution (peroxidase diluted 1:1000 with chromogenic substrate) were prepared (Beyotime, Shanghai, China). Cells were lysed with Western and IP cell lysis buffer. For the standard curve, 0, 12.5, 25, 50, or 75 μL of 5 mM H_2_O_2_ were adjusted to 100 μL with CAT detection buffer, yielding final concentrations of 0, 0.625, 1.25, 2.5, and 3.75 mM H_2_O_2_. Four microliters from each tube were added to a 96-well plate with 200 μL chromogenic working solution, incubated at 25 °C for ≥15 min, and the A_520_ was measured. For sample measurement, 6 μL of lysate was brought to 40 μL with CAT detection buffer, mixed with 10 μL of 250 mM H_2_O_2_, and reacted at 25 °C for 15 min. The reaction was stopped with 450 μL stop solution. Then 10 μL of the terminated mixture was diluted with 40 μL detection buffer, and 10 μL of this dilution was transferred to a 96-well plate with 200 μL chromogenic working solution. After incubation at 25 °C for 15–45 min, A_520_ was recorded. CAT activity was calculated from the standard curve (A_520_ = k × μmol H_2_O_2_ + b) as follows: consumed H_2_O_2_ (μmol) = (A_520_ − b)/k, and activity = [consumed μmol] × dilution factor/(reaction time in min × sample volume × protein concentration).

### 2.10. Total Superoxide Dismutase (SOD) Activity Detection

Cells were washed once with PBS and lysed with the provided SOD sample preparation solution (100–200 μL per 10^6^ cells). After centrifugation at 12,000× *g* for 3–5 min at 4 °C, the supernatant was collected as the sample. The WST-8/enzyme working solution (160 μL per reaction) was freshly prepared by mixing 151 μL SOD detection buffer, 8 μL WST-8, and 1 μL enzyme solution. The reaction initiation working solution was freshly prepared by diluting the 40× reaction initiator 1:40 with SOD detection buffer. Sample and blank wells were set up in a 96-well plate, and the reaction was initiated by adding the initiation working solution (Beyotime, Shanghai, China). Following incubation at 37 °C for 30 min, the absorbance at 450 nm was measured. The inhibition percentage was calculated as (A_blank1 − A_sample)/(A_blank1 − A_blank2) × 100%, and SOD activity was expressed as inhibition percentage/(1 − inhibition percentage) units.

### 2.11. RNA Extraction, cDNA Synthesis, and Quantitative Real-Time PCR

Total RNA was extracted from ovarian GCs using RNAiso Plus under RNase-free conditions. Cells were washed three times with PBS, lysed with 1 mL RNAiso Plus per well on ice for 15 min, and mixed with 200 μL chloroform. After centrifugation at 14,000× *g* for 10 min at 4 °C, the aqueous phase was transferred, mixed with an equal volume of isopropanol, incubated on ice for 5 min, and centrifuged again. The RNA pellet was washed three times with 500 μL ice-cold 75% ethanol, dried, dissolved in 15 μL RNase-free water, and quantified by spectrophotometry (A_260_/A_280_ ≥ 1.8).

One microgram of total RNA was treated with gDNA Eraser at 42 °C for 2 min to remove genomic DNA, and reverse transcription was performed using the PrimeScript™ RT Reagent Kit with gDNA Eraser (Takara, Kyoto, Japan) at 37 °C for 15 min, followed by enzyme inactivation at 85 °C for 5 s. Quantitative real-time PCR was carried out using TB Green Premix Ex Taq II (Takara, Kyoto, Japan) in a 10 μL reaction containing 2 μL cDNA and 0.4 μM each of forward and reverse primers. The thermal profile was 95 °C for 30 s, followed by 40 cycles of 95 °C for 5 s and 60 °C for 30 s. Melting curve analysis was performed to verify amplification specificity, and single-peak dissociation curves were obtained for all primer pairs. The amplification efficiency of each primer pair was determined by constructing standard curves from five 10-fold serial dilutions of pooled cDNA. All primer pairs exhibited amplification efficiencies between 90% and 110%, with correlation coefficients (R^2^) ≥ 0.99. The stability of β-actin as a reference gene across the Control, LPS, and LPS + Se groups was validated by comparing its Ct values; no statistically significant differences were observed among groups. Relative gene expression was calculated using the 2^−ΔΔCt^ method with β-actin as the internal control. Primers are listed in Table 1.

### 2.12. Western Blot Analysis

Total protein was extracted from ovine follicular GCs and quantified using a BCA protein assay kit. Separating and stacking gels were prepared according to the manufacturer’s instructions for the SDS-PAGE Gel Kit. Protein samples were mixed with loading buffer at a ratio of 1:4 (*v*/*v*), denatured at 100 °C for 10 min, and 10 μL of each sample was loaded per lane. Electrophoresis was performed at 200 V for 30 min. The gel regions containing target proteins were excised and transferred onto nitrocellulose (NC) membranes by wet transfer at 300 mA for 30 min. Membranes were blocked with rapid protein blocking buffer for 20 min at room temperature and subsequently incubated with primary antibodies overnight at 4 °C for 14 h. The following primary antibodies were used: anti-MFN1 (ABclonal, Wuhan, China, A15474) at 1:500, anti-OPA1 (ABclonal, A9833) at 1:4000, anti-P62 (ABclonal, Wuhan, China, A21702) at 1:2000, anti-DRP1 (Proteintech, Wuhan, China, 12957-1-AP) at 1:4000, anti-PARKIN (Proteintech, Wuhan, China, 14060-1-AP) at 1:2000, anti-BCL-2 (ABclonal, Wuhan, China, A0208SP) at 1:10,000, anti-BAX (ABclonal, Wuhan, China, A19684) at 1:40,000, anti-CASPASE3 (ABclonal, Wuhan, China, A19654) at 1:2000, and anti-β-actin (ABclonal, Wuhan, China, AC050) at 1:10,000. After three washes with TBST (10 min each), membranes were incubated with IRDye 800CW goat anti-rabbit secondary antibody (LI-COR, Nebraska, USA, 926-32211) at a 1:15,000 dilution for 90 min at room temperature, followed by three additional TBST washes. Protein bands were visualized using a gel imaging system, and band intensities were quantified with ImageJ software.

### 2.13. Statistical Analysis

All experiments were performed using three independent experimental replicates. Prior to statistical analysis, the normality of data distribution was assessed using the Kolmogorov–Smirnov test. Comparisons among multiple groups were performed using one-way analysis of variance (ANOVA) followed by Tukey’s honestly significant difference (HSD) post hoc test for multiple comparisons. All statistical analyses were conducted using SPSS (version 22.0; IBM, Armonk, NY, USA), and graphs were generated using GraphPad Prism (version 8.0; GraphPad Prism Software Inc., San Diego, CA, USA). Data are presented as mean ± standard error of the mean (SEM). Differences were considered statistically significant at *p* < 0.05, and highly significant at *p* < 0.01.

## 3. Results

### 3.1. Identification in Ovine GCs

**Figure 1:**
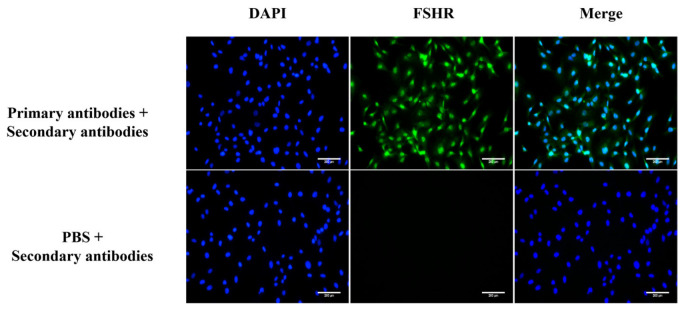
Immunofluorescence identification of ovine GCs. Immunofluorescence staining was performed to detect the expression of follicle-stimulating hormone receptor (FSHR, green), a specific marker of granulosa cells. Nuclei were counterstained with DAPI (blue). Merge represents the overlay of the two channels. Scale bar = 200 μm.

Immunofluorescence staining confirmed that FSHR was specifically expressed in the in vitro-cultured ovine GCs, with green fluorescence predominantly localized to the cell membrane and cytoplasm. DAPI counterstaining identified cell nuclei, confirming the nuclear localization of the fluorescence signal (Figure 1).

### 3.2. Establishment of the LPS-Induced Inflammatory Model

**Figure 2:**
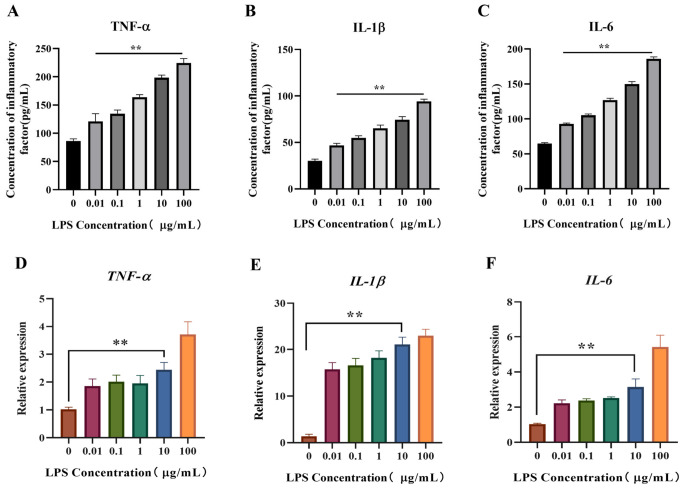
Construction of an LPS-induced inflammatory model of ovine ovarian GCs. (**A**–**C**): Concentrations of TNF-α, IL-1β, and IL-6 in the culture supernatant measured by ELISA; (**D**–**F**): Relative mRNA expression of *TNF-α*, *IL-1β*, and *IL-6* determined by qRT-PCR, with β-actin as the internal reference gene. Data are presented as mean ± SEM of three independent experimental replicates, ** *p* < 0.01 compared with the untreated control group (0 μg/mL LPS). Statistical analysis was performed using one-way ANOVA followed by Tukey’s HSD test.

**Figure 3:**
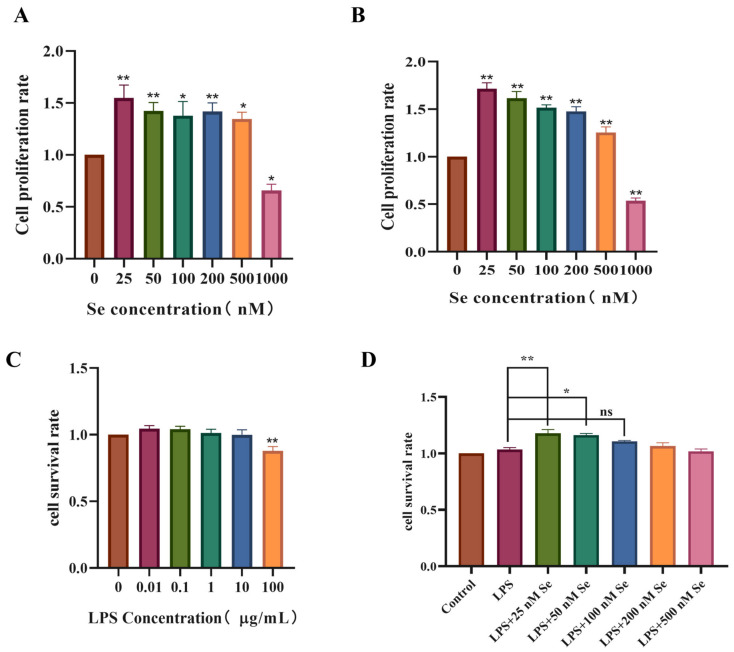
Screening of Se Concentration. (**A**): Cell viability after 24 h of treatment with different concentrations of sodium selenite (0–1000 nM); (**B**): Cell viability after 48 h of treatment with different concentrations of sodium selenite; (**C**): Cell viability of ovine GCs following treatment with different concentrations of LPS (0–100 μg/mL) for 12 h; (**D**): Cell viability of LPS-injured ovine GCs after treatment with different concentrations of sodium selenite (25–500 nM). Data are presented as mean ± SEM of three independent experimental replicates. * *p* < 0.05, ** *p* < 0.01 compared with the untreated control group (0 nM or 0 μg/mL)*,* ns: not significant. Statistical analysis was performed using one-way ANOVA followed by Tukey’s HSD test.

To establish an LPS-induced inflammatory model, ovine ovarian GCs were treated with different concentrations of LPS (0, 0.01, 0.1, 1, 10, and 100 μg/mL) for 12 h, ELISA results demonstrated that treatment with 10 μg/mL LPS for 12 h significantly increased the secretion of TNF-α, IL-6, and IL-1β in the cell supernatant (*p* < 0.01, Figure 2B–D), indicating successful induction of the inflammatory response. qRT-PCR analysis further confirmed that the mRNA expression levels of *TNF-α*, *IL-6*, and *IL-1β* were significantly upregulated in the LPS-treated group (*p* < 0.01, Figure 2E,F). Cell viability was assessed by the CCK-8 assay. No significant difference in cell viability was observed between the 10 μg/mL LPS group and the control group (*p* > 0.05), whereas 100 μg/mL LPS significantly inhibited cell proliferation (*p* < 0.05, Figure 3C). Since 10 μg/mL LPS for 12 h effectively activated the inflammatory response with minimal impact on cell viability, this condition was selected as optimal for subsequent experiments.

### 3.3. Screening for the Optimal Se Concentration

To determine the optimal Se concentration, cell viability was assessed by the CCK-8 assay after treatment with different concentrations of sodium selenite (0, 25, 50, 100, 200, 500, and 1000 nM) for 24 h (Figure 3A) and 48 h (Figure 3B). The results showed that 25–500 nM sodium selenite significantly enhanced cell viability compared with the control group at both time points (*p* < 0.05 or *p* < 0.01). Cell viability decreased as the Se concentration increased, with the peak value observed at 25 nM, and this trend was consistent at both 24 and 48 h. In LPS-injured GCs, cell viability was significantly higher in the 25 and 50 nM LPS + Se groups than in the LPS alone group (*p* < 0.05 or *p* < 0.01), with the 25 nM group exhibiting the optimal protective effect. Therefore, 25 nM Se was selected for all subsequent experiments.

### 3.4. Effects of Se on the Morphological Structure of LPS-Induced Ovine Ovarian GCs

**Figure 4:**
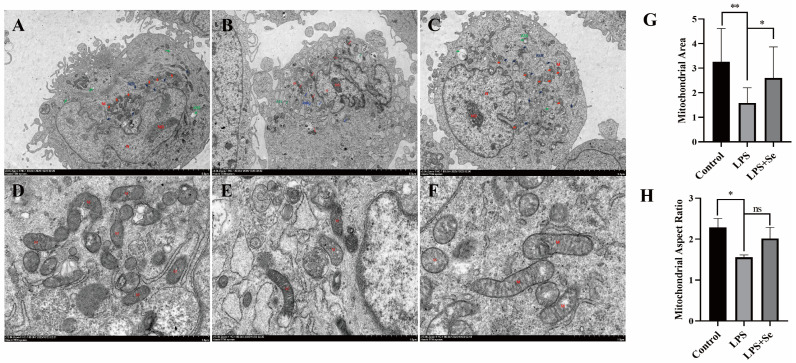
Effects of Se on the morphological structure of LPS-induced ovine ovarian follicular GCs. (**A**–**C**): Representative TEM images of the Control (**A**), LPS (**B**), and LPS + Se (**C**) groups; (**D**–**F**): Partially magnified images of the corresponding groups showing detailed mitochondrial ultrastructure; (**G**): Quantification of mitochondrial area; (**H**): Quantification of mitochondrial aspect ratio. In panels (**A**–**F**), N indicates nucleus; No, nucleolus; red arrows, mitochondria (M); blue arrows, rough endoplasmic reticulum (RER); green arrows, autophagolysosomes (ASS). Data are presented as mean ± SEM of three independent experimental replicates. * *p* < 0.05, ** *p* < 0.01, ns: no significant difference (*p* > 0.05). Statistical analysis was performed using one-way ANOVA followed by Tukey’s HSD test.

TEM images (Figure 4A–F) revealed that, compared with the control group, GCs in the LPS-treated group exhibited marked ultrastructural abnormalities, including mitochondrial pyknosis, reduced cristae numbers and thickened cristae, increased membrane density, and pronounced dilation of the rough endoplasmic reticulum. Autophagolysosomes and a few vacuoles were also observed. In contrast, the LPS + Se group showed substantially improved mitochondrial morphology, with most mitochondria returning to an oval shape and displaying clearer, more organized cristae, suggesting a protective effect of Se against LPS-induced ultrastructural damage.

To quantitatively evaluate this protective effect, mitochondrial area and aspect ratio were measured from TEM images. Compared with the Control group, LPS treatment significantly reduced the mean mitochondrial area (*p* < 0.01, Figure 4G), indicating pronounced mitochondrial shrinkage. Se supplementation in the LPS + Se group significantly increased the mitochondrial area relative to the LPS group (*p* < 0.05, Figure 4G), indicating that Se effectively ameliorated the LPS-induced decline in mitochondrial area. The mitochondrial aspect ratio, defined as the ratio of the major axis to the minor axis, serves as a key indicator of mitochondrial fragmentation, with lower values reflecting a fragmented morphology. LPS treatment significantly decreased the mitochondrial aspect ratio compared with the Control group (*p* < 0.05, Figure 4H), indicative of excessive mitochondrial fragmentation. Notably, the aspect ratio in the LPS + Se group was significantly higher than that in the LPS group and did not differ statistically from the Control group (*p* > 0.05, Figure 4H). These quantitative data suggest that Se supplementation attenuates LPS-induced mitochondrial fragmentation and helps preserve mitochondrial morphological integrity. However, we acknowledge that these ultrastructural observations remain largely descriptive and that the sample size for quantification is limited. Further studies with larger sample sizes and additional morphometric parameters are warranted to strengthen these findings.

### 3.5. Effects of Se on ΔΨm, Mitophagy-Related Markers, and Mitochondrial Dynamics in LPS-Induced Ovine Follicular GCs

To evaluate the protective effect of Se against LPS-induced mitochondrial injury in ovine follicular GCs, ΔΨm, mitophagy-related markers, and mitochondrial dynamics-related proteins were assessed using JC-1 staining, qRT-PCR, and Western blot. JC-1 staining showed that the CCCP-treated positive control group exhibited predominantly green fluorescence (JC-1 monomers) with negligible red fluorescence (JC-1 aggregates), confirming complete mitochondrial depolarization. The control group displayed bright red fluorescence and weak green fluorescence, indicating normal ΔΨm. Compared with the control group, the LPS-treated group showed markedly attenuated red fluorescence and enhanced green fluorescence, suggesting a pronounced LPS-induced decrease in ΔΨm. In contrast, LPS + Se co-treatment substantially restored red fluorescence and reduced green fluorescence, indicating that Se effectively protected against LPS-induced ΔΨm collapse.

**Figure 5:**
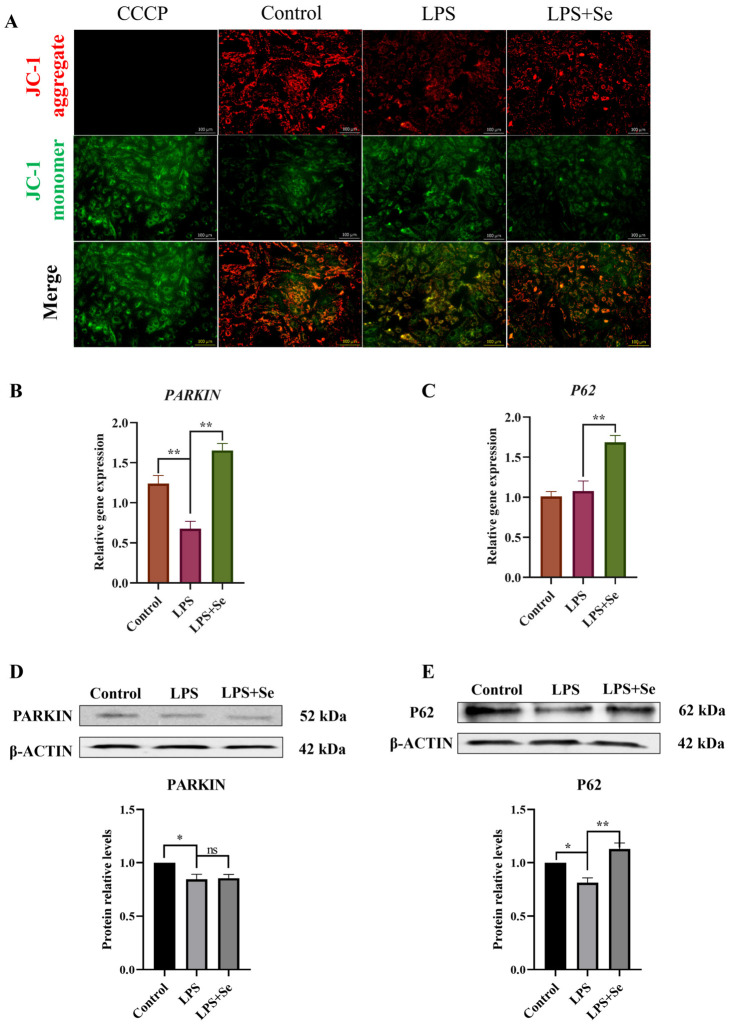
Effects of Se on ΔΨm and PARKIN/P62 expression in LPS-induced ovine GCs. (**A**): ΔΨm assessed by JC-1 staining. Red fluorescence (JC-1 aggregates) indicates high ΔΨm, green fluorescence (JC-1 monomers) indicates low ΔΨm. CCCP was used as a positive control for mitochondrial depolarization. Scale bar = 100 μm. (**B**,**C**): Relative mRNA expression of *PARKIN* (**B**) and *P62* (**C**) quantified by qRT-PCR, with β-actin as the internal reference gene. (**D**,**E**): Representative Western blot images and quantification of PARKIN (**D**) and P62 (**E**) protein levels. Data are presented as mean ± SEM of three independent experimental replicates. * *p* < 0.05, ** *p* < 0.01; ns, not significant (*p* > 0.05). Statistical analysis was performed using one-way ANOVA followed by Tukey’s HSD test.

qRT-PCR and Western blot analyses revealed that LPS treatment significantly downregulated both the mRNA (*p* < 0.01, Figure 5B) and protein (*p* < 0.05, Figure 5D) levels of the mitophagy-associated protein PARKIN, and significantly decreased the protein level of P62 (*p* < 0.05, Figure 5E), whereas *P62* mRNA expression was not significantly affected (*p* > 0.05, Figure 5C). Compared with the LPS alone group, LPS + Se co-treatment significantly upregulated *PARKIN* mRNA (*p* < 0.01, Figure 5B) and *P62* mRNA (*p* < 0.01, Figure 5C), and significantly restored P62 protein expression (*p* < 0.01, Figure 5E); PARKIN protein expression showed an upward trend that did not reach statistical significance (*p* > 0.05, Figure 5D).

**Figure 6:**
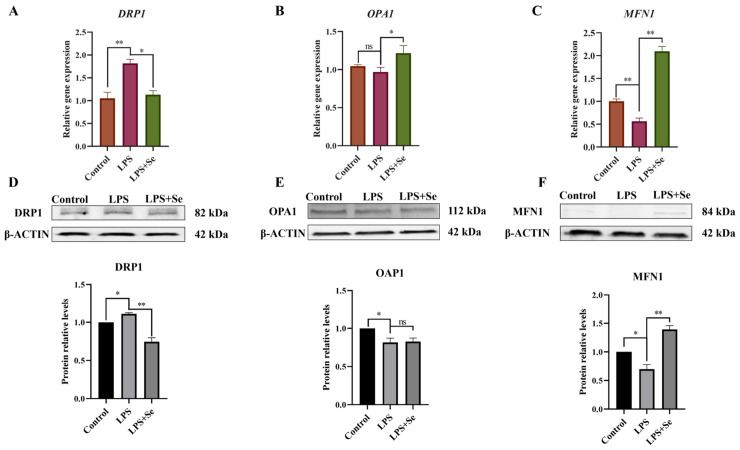
Effects of Se on mitochondrial dynamics-related gene and protein expression in LPS-induced ovine follicular GCs. (**A**–**C**): Relative mRNA expression of the mitochondrial fission gene DRP1 (**A**) and fusion genes OPA1 (**B**) and MFN1 (**C**) determined by qRT-PCR, with β-actin as the internal reference gene. (**D**–**F**): Representative Western blot images and quantification of DRP1 (**D**), OPA1 (**E**), and MFN1 (**F**) protein levels. β-actin served as the loading control. All data are presented as mean ± SEM of three independent experimental replicates. * *p* < 0.05, ** *p* < 0.01; ns: not statistically significant. Statistical analysis was performed using one-way ANOVA followed by Tukey’s HSD test.

With respect to mitochondrial dynamics, LPS treatment significantly upregulated the mRNA (*p* < 0.01, Figure 6A) and protein (*p* < 0.05, Figure 6D) levels of the fission protein DRP1, and significantly downregulated the mRNA (*p* < 0.01, Figure 6C) and protein (*p* < 0.05, Figure 6F) levels of the fusion protein MFN1, as well as the protein level of OPA1 (*p* < 0.05, Figure 6B); *OPA1* mRNA expression was not significantly altered (*p* > 0.05, Figure 6E). Conversely, LPS + Se co-treatment significantly downregulated *DRP1* mRNA (*p* < 0.05, Figure 6A) and protein (*p* < 0.01, Figure 6D), and significantly upregulated *MFN1* mRNA (*p* < 0.01, Figure 6C) and protein (*p* < 0.01, Figure 6E), as well as *OPA1* mRNA (*p* < 0.05, Figure 6B); OPA1 protein expression showed an upward trend that did not reach statistical significance (*p* > 0.05, Figure 6E). These findings indicate that Se alleviates LPS-induced mitochondrial injury in ovine GCs, which is associated with the modulation of PARKIN and P62 expression and the regulation of mitochondrial fission and fusion proteins.

### 3.6. Effects of Se on the Expression of Inflammatory Factors in LPS-Induced Ovine Ovarian Follicular GCs

**Figure 7:**
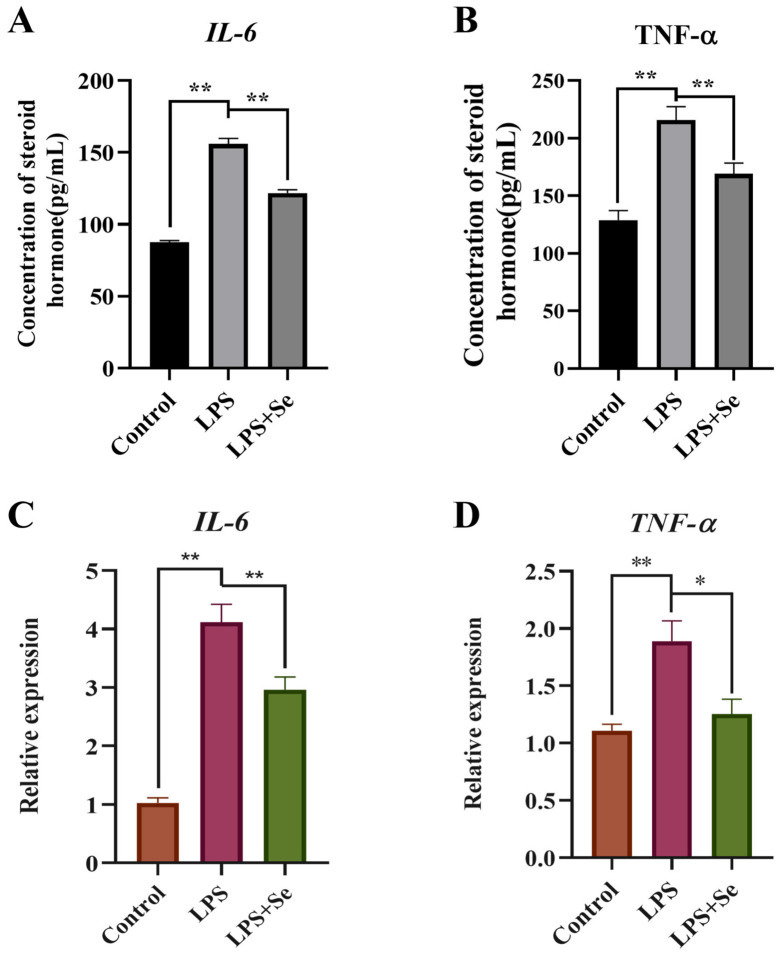
Effects of Se on the expression of inflammatory factors in LPS-induced ovine ovarian follicular GCs. (**A**,**B**): Concentrations of IL-6 (**A**) and TNF-α (**B**) in the culture supernatant measured by ELISA; (**C**,**D**): Relative mRNA expression of *IL-6* (**C**) and *TNF-α* (**D**) determined by qRT-PCR, with β-actin as the internal reference gene. Data are presented as mean ± SEM of three independent experimental replicates. * *p* < 0.05, ** *p* < 0.01. Statistical analysis was performed using one-way ANOVA followed by Tukey’s HSD test.

After Se intervention, ELISA results showed that the secretion levels of IL-6 and TNF-α were significantly lower than those in the LPS model group (*p* < 0.01, Figure 7A,B). qRT-PCR analysis further demonstrated that Se intervention significantly downregulated the mRNA expression of *IL-6* and *TNF-α* compared with the LPS group (*p* < 0.01, Figure 7C,D), indicating that Se effectively alleviated the LPS-induced inflammatory response.

### 3.7. Effects of Se on the Proliferation and Apoptosis of LPS-Induced Ovine Ovarian Follicular GCs

**Figure 8:**
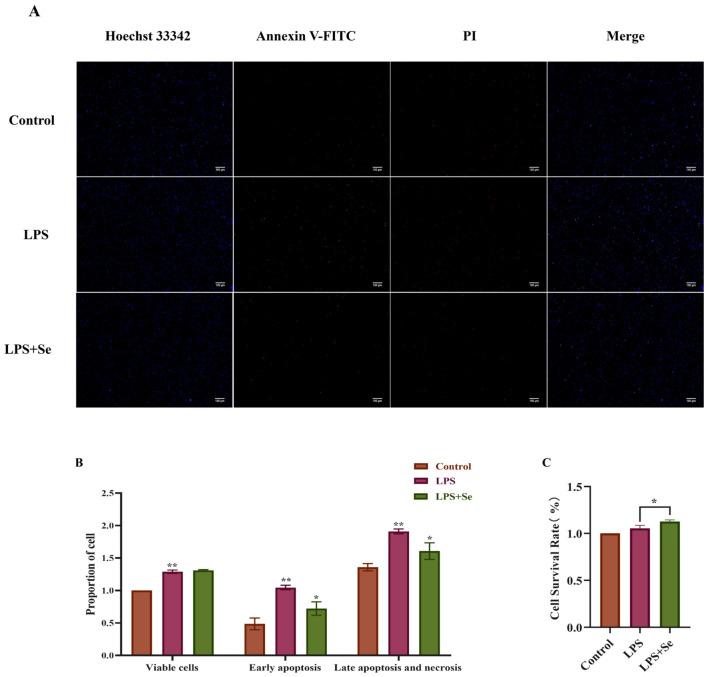
Effects of Se on the proliferation and apoptosis of LPS-induced ovine ovarian follicular GCs. (**A**): Representative fluorescence images of Annexin V-FITC/PI staining. Blue fluorescence (Hoechst 33342) indicates all cell nuclei; Green fluorescence (Annexin V-FITC-positive) indicates early apoptosis; red fluorescence (PI-positive) indicates late apoptosis or necrosis. Scale bar = 100 μm; (**B**): Quantification of the apoptotic rate (early + late apoptosis); (**C**): Cell viability assessed by the CCK-8 assay. Data are presented as mean ± SEM of three independent experimental replicates. * *p* < 0.05, ** *p* < 0.01. Statistical analysis was performed using one-way ANOVA followed by Tukey’s HSD test.

**Figure 9:**
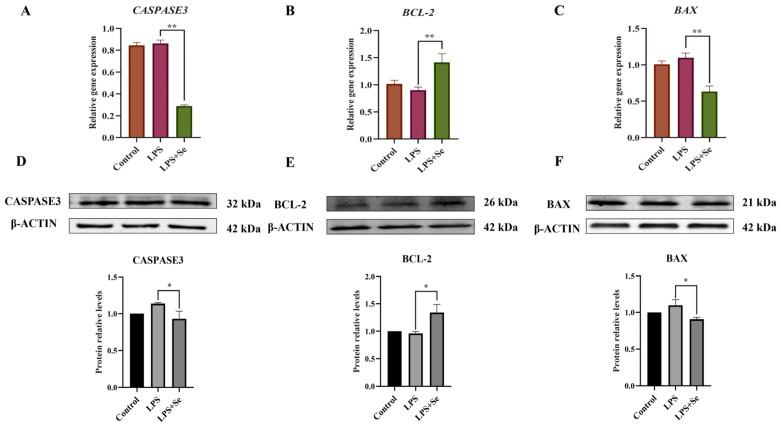
Effects of Se on apoptosis-related gene and protein expression in LPS-induced ovine follicular GCs. (**A**–**C**): Relative mRNA expression of *CASPASE3* (**A**), *BCL-2* (**B**), and *BAX* (**C**) determined by qRT-PCR, with β-actin as the internal reference gene; (**D**–**F**): Representative Western blot images and quantification of CASPASE3 (**D**), BCL-2 (**E**), and BAX (**F**) protein levels. β-actin served as the loading control; Data are presented as mean ± SEM of three independent experimental replicates. * *p* < 0.05, ** *p* < 0.01. Statistical analysis was performed using one-way ANOVA followed by Tukey’s HSD test.

The CCK-8 assay showed that the cell viability in the Se intervention group was significantly higher than that in the LPS model group (*p* < 0.05, Figure 8C), indicating that Se effectively restored the viability of LPS-injured ovine follicular GCs. Annexin V-FITC/PI staining revealed that early apoptotic cells were Annexin V-FITC-positive and PI-negative, whereas late apoptotic or necrotic cells were double-positive. LPS treatment significantly increased both the early and late apoptotic rates compared with the control group, while Se intervention significantly reduced these rates (*p* < 0.05 or *p* < 0.01, Figure 8A,B), suggesting an anti-apoptotic effect of Se. To further investigate the underlying mechanism, the mRNA and protein expression of CASPASE3, BAX, and BCL-2 were examined. qRT-PCR results showed that LPS significantly upregulated *CASPASE3* and *BAX* mRNA expression and downregulated *BCL-2* mRNA expression compared with the control group (*p* < 0.01, Figure 9A–C). LPS + Se significantly reversed these changes (*p* < 0.01, Figure 9A–C). Western blot analysis confirmed consistent trends at the protein level (Figure 9D–F).

### 3.8. Effects of Se on the Antioxidant Capacity of LPS-Induced Ovine Ovarian Follicular GCs

**Figure 10:**
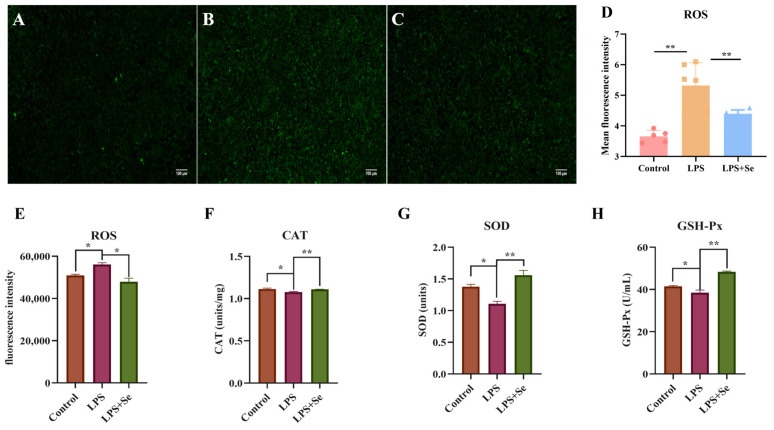
Effects of Se on the antioxidant capacity of LPS-induced ovine ovarian GCs. (**A**–**C**): Representative fluorescence images of intracellular ROS detected by DCFH-DA staining (green) in the Control (**A**), LPS (**B**), and LPS + Se (**C**) groups. Scale bar = 100 μm; (**D**): Quantification of ROS fluorescence intensity; (**E**): Quantitative detection of relative ROS levels; (**F**): CAT activity; (**G**): SOD activity; (**H**): GSH-Px activity. Data are presented as mean ± SEM of three independent experimental replicates. * *p* < 0.05, ** *p* < 0.01. Statistical analysis was performed using one-way ANOVA followed by Tukey’s HSD test.

As shown in Figure 10A–D, LPS treatment significantly increased intracellular ROS levels (*p* < 0.01), whereas Se intervention markedly reduced ROS accumulation (*p* < 0.01), confirming the antioxidant capacity of Se. The activities of the antioxidant enzymes CAT, SOD and GSH-Px were also measured (Figure 10F–H). LPS treatment significantly decreased the activities of all three enzymes (*p* < 0.05). Compared with the LPS group, the LPS + Se group exhibited significantly restored enzyme activities (*p* < 0.05 or *p* < 0.01). CAT activity returned to near-control levels, while GSH-Px and SOD activities were even higher than those in the control group. These results demonstrate that Se not only effectively scavenged LPS-induced excessive ROS but also enhanced the activity of the endogenous antioxidant enzyme system, thereby contributing to the maintenance of redox homeostasis.

### 3.9. Effects of Se on Steroid Hormones in LPS-Induced Ovine Ovarian Follicular GCs

**Figure 11:**
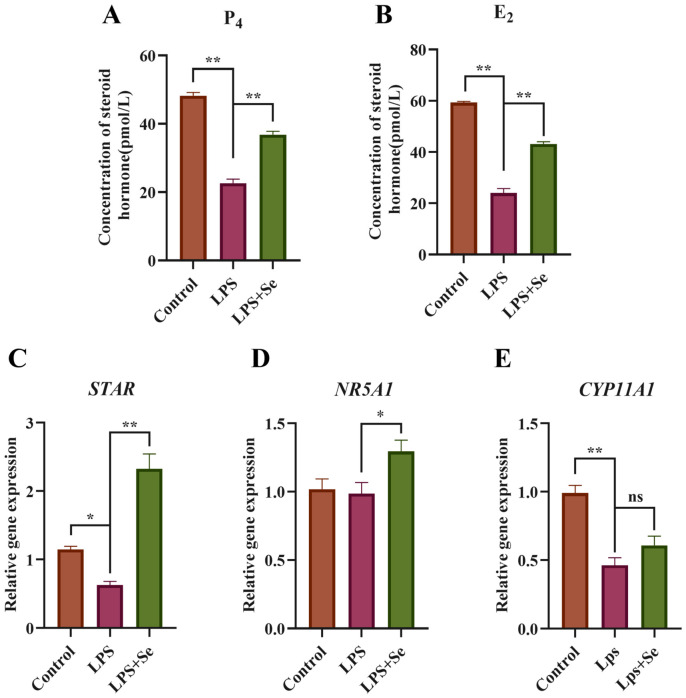
Effects of Se on steroid hormone synthesis in LPS-induced ovine ovarian follicular GCs. (**A**,**B**): Secretion levels of estradiol (E_2_) (**A**) and progesterone (P_4_) (**B**) in the culture supernatant measured by ELISA; (**C**–**E**): Relative mRNA expression of NR5A1 (**C**), STAR (**D**), and CYP11A1 (**E**) determined by qRT-PCR, with β-actin as the internal reference gene. Data are presented as mean ± SEM of three independent experimental replicates; * *p* < 0.05, ** *p* < 0.01, ns: not significant. Statistical analysis was performed using one-way ANOVA followed by Tukey’s HSD test.

ELISA results showed that LPS treatment significantly inhibited the secretion levels of E_2_ and P_4_ in ovine ovarian GCs (*p* < 0.01), while Se intervention significantly enhanced the synthesis and release of E_2_ and P_4_ (*p* < 0.05 or *p* < 0.01, Figure 11A,B). qPCR analysis further revealed that Se significantly upregulated the expression levels of nuclear receptor subfamily 5 group A member 1 (*NR5A1*) and steroidogenic acute regulatory protein (*STAR*) mRNA (*p* < 0.05 or *p* < 0.01, Figure 11C,D). whereas cytochrome P450 family 11 subfamily A member 1 (*CYP11A1*) mRNA expression showed an increasing trend that did not reach statistical significance (Figure 11E). These findings suggest that Se partially restored E_2_ and P_4_ secretion, which was associated with the upregulation of STAR and NR5A1 mRNA expression. However, a more comprehensive assessment of additional steroidogenic enzymes is warranted to fully elucidate the underlying mechanisms.

## 4. Discussion

### 4.1. Effects of Se on Ovine Follicular GCs

Ovarian follicular development is a tightly orchestrated process that determines sheep reproductive performance [25], and GCs are core somatic cells within the follicle, responsible for supporting oocyte maturation by secreting steroid hormones and maintaining follicular microenvironment homeostasis [26]. Dysregulation of GC function, especially under inflammatory or oxidative stress conditions, is a key driver of follicular atresia. Se, an essential trace element for maintaining homeostasis in humans and animals [27], cannot be endogenously synthesized and must be acquired through dietary intake. However, ruminants exhibit suboptimal Se absorption and utilization due to factors including Se speciation, dosage, and interactions with other trace elements [28]. Appropriately dosed Se supplementation modulates metabolic disorders, enhances cellular survival, and improves immunocompetence, thereby supporting overall health and reproductive performance in livestock [29]. In the present study, supplementation with 25–500 nM Se promoted the proliferation of ovine GCs in vitro, and 25 nM Se exhibited the optimal protective effect in alleviating LPS-induced GC injury.

### 4.2. LPS-Induced Inflammatory Model of Ovine Ovarian Follicular GCs

GC inflammation is a pivotal driver of ovarian functional decline—manifested as follicular atresia and luteal insufficiency—that directly impairs steroidogenesis and oocyte maturation. LPS, an endotoxin component of Gram-negative bacterial cell walls, serves as a well-established in vitro inflammatory inducer [30] due to its high efficiency and stability in triggering inflammatory reactions. In this study, treatment with 10 μg/mL LPS for 12 h not only preserved cellular viability but also effectively simulated pathological inflammation, thereby providing a suitable platform to investigate the regulatory role of Se in GC inflammation. TNF-α is a pleiotropic pro-inflammatory cytokine that triggers extrinsic apoptosis and necroptosis [31]. IL-6, produced by monocytes, fibroblasts, endothelial cells, and lymphocytes during inflammatory insults, activates the gp130/IL-6R complex to stimulate NF-κB signaling, amplifying inflammatory cascades [32]. IL-6 overexpression exacerbates ovarian dysfunction in LPS-challenged murine models, whereas its suppression alleviates pathology [33]. Our findings demonstrated that LPS significantly induced the expression of TNF-α, IL-1β, and IL-6 in ovine GCs, while Se pretreatment significantly inhibited the LPS-induced upregulation of TNF-α and IL-6, indicating that Se effectively alleviated the LPS-induced inflammatory response. Se supplementation has also been reported to reduce the incidence of postpartum diseases in female livestock, such as metritis, retained placenta, and ovarian cysts, and to improve the conception rate of dairy cows [34].

### 4.3. Se Regulated Mitophagy and Mitochondrial Dynamic Homeostasis in LPS-Induced Ovine Follicular GCs

Mitochondria, as the central hubs of energy metabolism in eukaryotic cells, rely on structural and functional integrity to sustain follicular granulosa cell proliferation, differentiation, and steroidogenesis; conversely, mitochondrial homeostatic imbalance represents a core mechanism underlying inflammation-induced ovarian dysfunction [35]. Mitophagy serves as a critical quality-control mechanism for the selective elimination of damaged mitochondria, with the PARKIN–PINK1 pathway being the most extensively characterized mitophagic regulatory axis [36]. Mitochondrial fission and fusion, as essential components of mitochondrial quality control, constitute two opposing yet coordinated processes that collectively govern mitochondrial morphology, number, and function [37]. The present study provides evidence for the regulatory role of Se in mitochondrial ultrastructure and the expression of mitophagy- and dynamics-related proteins in ovine follicular GCs under LPS-induced inflammatory conditions. Our results demonstrate that LPS exposure triggers cristae structural disruption, morphological fragmentation, a significant reduction in mitochondrial area, and a pronounced decrease in aspect ratio, accompanied by loss of ΔΨm, downregulation of PARKIN and P62 protein expression, and a dynamics imbalance characterized by enhanced fission via upregulated DRP1 and suppressed fusion via downregulated MFN1 and OPA1. Se supplementation effectively reversed these pathological alterations by upregulating the mRNA expression of PARKIN and P62, while bidirectionally modulating the expression of fission and fusion proteins (downregulating DRP1; upregulating MFN1 and OPA1), thereby contributing to the preservation of mitochondrial ultrastructural integrity and membrane potential. However, it is important to note that the present study assessed only the expression levels of PARKIN and P62. Direct indicators of functional mitophagic flux—such as the LC3-II/LC3-I ratio, PINK1 accumulation, lysosomal activity, and fluorescence-based mitophagy imaging—were not evaluated. Therefore, the observed changes in PARKIN and P62 protein levels suggest a modulatory effect of Se on mitophagy-associated markers rather than a definitive restoration of mitophagic flux. Future studies incorporating autophagic flux inhibitors such as chloroquine or bafilomycin A1 and mitophagy detection assays will be necessary to establish the functional role of Se in mitophagy regulation. Although the increases in PARKIN and OPA1 protein expression did not reach statistical significance, both exhibited a clear upward trend, which may be attributable to the timing of protein detection or to the existence of multiple OPA1 splice variants subject to post-translational regulation. A close reciprocal regulatory relationship exists between mitophagy and mitochondrial dynamics: mitochondrial fission maintains mitochondrial number and distribution, with excessively fragmented mitochondria serving as preferential substrates for mitophagy, whereas mitochondrial fusion sustains normal mitochondrial function through content exchange between fusing mitochondria [38]. The capacity of Se to concurrently modulate both processes in this study suggests that it may orchestrate holistic regulation of mitochondrial homeostasis through a common upstream signaling pathway, and that this protective effect is intimately associated with Se-mediated suppression of inflammatory pathway activation. As an essential cofactor of glutathione peroxidase (GPx), Se can alleviate lipid peroxidative damage by scavenging mitochondrial ROS, which also constitutes a key molecular basis for its role in maintaining mitochondrial membrane stability [39]. Although this study provides novel experimental evidence for the ovarian protective mechanisms of Se, several limitations should be acknowledged. First, all findings were obtained exclusively at the in vitro cellular level, and the precise molecular mechanisms by which Se regulates PARKIN and DRP1 expression, as well as its comprehensive impact on mitochondrial energy metabolism—including ATP production, oxygen consumption rate, and mtDNA copy number—remain to be elucidated. Additionally, mitochondrial aspect ratios were derived from two-dimensional TEM images, which represent planar projections of three-dimensional structures. Although conservative selection criteria (mitochondria with unambiguous membranes and clearly resolved cristae) and blinded random sampling were employed to minimize orientation bias, the measured values should be interpreted as estimates of mitochondrial elongation rather than absolute three-dimensional parameters. Future studies using serial-section TEM or electron tomography could provide more comprehensive morphological data. Despite these limitations, the present findings offer a theoretical reference for the nutritional prevention and control of inflammatory ovarian diseases in livestock production.

### 4.4. Effects of Se on Oxidative Stress Injury in LPS-Induced Ovine GCs

As an essential component of multiple antioxidant enzymes, Se suppresses ROS generation and plays a pivotal role in protecting cells against oxidative damage [40]. LPS challenge triggers pathological cascades including inflammation, oxidative stress, and apoptosis [41]. Antioxidant enzymes such as SOD and GSH-Px constitute the primary cellular defense against free radicals [42]. The decomposition of H_2_O_2_ to H_2_O and O_2_ by peroxidases represents a fundamental free radical-scavenging mechanism that maintains redox homeostasis and preserves cellular function [43]. In the present study, Se intervention reversed LPS-induced decreases in SOD, GSH-Px, and CAT activities and attenuated ROS accumulation, highlighting its ability to bolster antioxidant defenses and protect against oxidative injury. Mechanistically, Se restored antioxidant enzyme activity, maintained redox equilibrium, and synergized with its anti-inflammatory and anti-apoptotic functions, thereby establishing a multi-pathway protective network against cellular dysfunction. This integrated effect enhanced ovine follicular GC viability under inflammatory conditions, underscoring the therapeutic potential of Se in regulating reproductive cell homeostasis.

### 4.5. Effects of Se on Proliferation and Apoptosis in LPS-Induced Ovine Ovarian Follicular GCs

In mammalian ovaries, a tightly regulated balance between cellular proliferation and apoptosis is essential for GC homeostasis [44]. Apoptosis is orchestrated by the BCL-2 and CASPASE protein families: BCL-2 counteracts the pro-apoptotic protein BAX to preserve mitochondrial integrity, while CASPASE-3 executes the apoptotic cascade [45]. Although inflammation serves as a physiological defense mechanism, dysregulated responses cause severe tissue damage [46]. In ovarian contexts, inflammatory insults disrupt the follicular microenvironment, triggering follicular atresia and granulosa cell dysfunction, compromising oocyte quality, and establishing a synergistic apoptosis–inflammation interplay that accelerates ovarian functional decline. Our data demonstrate that Se intervention reversed LPS-induced apoptosis by upregulating BCL-2 expression and downregulating BAX and CASPASE-3 transcription. As an essential trace element, Se promotes granulosa cell proliferation [47] and oocyte maturation, consistent with its proliferative effects in caprine luteinized GCs [48]. We acknowledge that the quantification of apoptosis in the present study relied on fluorescence microscopy and manual counting using ImageJ software. Flow cytometry, which provides more robust, automated, and high-throughput single-cell quantification, is the gold standard for Annexin V/PI analysis, and its absence represents a methodological limitation of the current study. Future investigations should employ flow cytometric validation to further strengthen the apoptosis data. Collectively, these findings suggest that Se not only mitigates inflammation but also confers robust anti-apoptotic protection to ovine follicular GCs by modulating the key apoptotic mediators, thereby helping to maintain cellular viability under inflammatory conditions.

### 4.6. Effects of Se on Steroid Hormone Synthesis in LPS-Induced Ovine Ovarian Follicular GCs

Follicular development critically determines female reproductive performance and health, and is orchestrated by the hypothalamic-pituitary-ovarian axis [49]. P_4_ and E_2_ are tetracyclic lipid hydrocarbons derived from cholesterol that play pivotal roles in female reproductive function and folliculogenesis [50]. STAR plays a crucial role in transporting cholesterol to the inner mitochondrial membrane [51], where CYP11A1 catalyzes the conversion of mitochondrial cholesterol into pregnenolone, initiating steroid synthesis [52]. *NR5A1* is a master regulatory gene that regulates the expression of numerous genes encoding reproductive and steroidogenic enzymes in sheep GCs [53]. During disease states, LPS accumulates in follicular fluid, triggering GC inflammation via TLR4 signaling. Uterine-diseased dairy cows exhibit impaired follicular growth and reduced plasma E_2_, and LPS suppresses E_2_ synthesis in bovine GCs [9]. Our key experimental findings revealed that LPS treatment significantly inhibited E_2_ and P_4_ secretion in ovine follicular GCs. Se intervention, by contrast, substantially increased E_2_ and P_4_ secretion, and upregulated the mRNA expression of *NR5A1* and *STAR*, suggesting that Se partially restored steroidogenesis. This observation aligns with prior evidence demonstrating that sodium selenite attenuates H_2_O_2_-induced ROS elevation in bovine GCs, suppresses apoptosis, and upregulates STAR expression, thereby effectively reversing hormonal suppression [54]. However, we acknowledge that the present study assessed the mRNA expression of only three steroidogenic genes (STAR, NR5A1, and CYP11A1). Additional key enzymes, such as aromatase (CYP19A1) and 3β-hydroxysteroid dehydrogenase (HSD3B), were not evaluated. A more comprehensive analysis of steroidogenic pathway enzymes is warranted in future investigations to fully elucidate the mechanisms underlying Se-mediated regulation of hormone synthesis. Collectively, these results suggest that Se promotes follicular development, mitigates GC damage, and helps preserve endocrine function under oxidative and inflammatory stress.

This study has several limitations. First, all findings were obtained from in vitro-cultured ovine ovarian follicular GCs, providing only in vitro cytological evidence without in vivo animal model validation; the applicability of our conclusions under in vivo physiological conditions remains to be confirmed. Second, the absence of a Se-only control group precludes the distinction between Se-mediated reversal of LPS-induced injury and its potential independent effects. Although previous studies have reported that Se can promote granulosa cell proliferation and steroidogenesis under basal conditions, the present study was specifically designed to investigate the protective effect of Se against LPS-induced damage. The independent effects of Se treatment alone will be evaluated in future investigations. Third, the number of mitochondria analyzed per group for quantitative TEM was relatively limited. Future studies will establish an LPS-induced in vivo model of ovine endometritis or oophoritis to systematically investigate the effect of dietary Se on ovarian mitochondrial ultrastructure and its targeted regulatory mechanisms, thereby providing more comprehensive experimental evidence for the application of Se in regulating sheep reproductive performance.

## 5. Conclusions

Our findings indicate that mitochondrial structural disruption is a critical pathological feature of LPS-induced ovine GC injury. Se ameliorated this damage by preserving mitochondrial ultrastructure and partially restoring membrane potential, effects that were associated with an improved balance between proliferation and apoptosis, enhanced antioxidant defense, suppressed inflammation, and partially restored steroid hormone secretion. These in vitro findings suggest that Se-mediated preservation of mitochondrial structure contributes to its protective effects in ovine GCs. However, further in vivo studies are required to validate these observations and to evaluate the potential application of Se as a nutritional strategy for mitigating inflammation-driven follicular atresia in ruminants.

## Figures and Tables

**Table 1:** Primer sequences of qRT-PCR.

Gene	PRIMER SEQUENCE	LENGTH	ACCESSION
*BAX*	F: GACATTGGACTTCCTTCGAGA R: AGCACTCCAGCCACAAAGAT	126	XM_027978592.3
*BCL-2*	F: GTGGATGACCGAGTACCTGAAC R: AGACAGCCAGGAGAAATCAAAC	124	XM_012103831.5
*CASPASE3*	F: CCCACTGCAGCAACATTAATCC R: CGACAGGCCATGCCAGTATT	252	XM_060406952.1
*CYP11A1*	F: AGGCAGAGGGAGACATAAGCA R: GTGTCTTGGCAGGAATCAGGT	156	XM_060401375.1
*NR5A1*	F: AGGAGGTGCGGGGCTC R: GCTTGAAGAAGCCCTTGCAG	190	XM_042245715.2
*STAR*	F: CAGAAGGGTGTCATCAGAGCG R: CAAAATCCACCTGGGTCTGC	169	XM_060406892.1
*TNF-α*	F: TTGTTCCTCACCCACACCAT R: GGCAGAGAGGATGTTGACCT	69	NM_001024860.1
*IL-6*	F: ATCTGGGTTCAATCAGGCGAT R: GCTCTGCAACTCCATGACAG	127	NM_001009392.1
*IL-1β*	F: CATGTGTGCTGAAGGCTCTC R: CAGGGTCGGTGTATCACCTT	173	NM_001009465.2
*P62*	F: GCCTTTCATCCAGAAGCTGT R: TCATCACCTGGCTCCTCTTC	91	NM_001009784.3
*PARKIN*	F: AAGACCTCTACGCCAACACG R: GCCAGGGCAGTGATCTCTTT	190	XM_027956386.2
*MFN1*	F: CCAGCAACGCCAGATAATGC R: TCCAACAACGATGATGCCCA	183	XM 015104932.3
*OPA1*	F: CCTGACATCGTGTGGGAGAT R: TCCAGGTGAACCTGTGGTG	79	XM_004014953.4
*DRP1*	F: GTGGGAACGCAGAGCAGT R: TGCTTCAACTCCATTTTCTTCTCC	174	XM 042253359.1
*β-ACTIN*	F: GGATGAGGCTCAGAGCAAGAGA R: TCGTCCCAGTTGGTGACGAT	78	U39357.2

## Data Availability

The original contributions presented in the study are included in the article, further inquiries can be directed to the corresponding authors.

## References

[1] Zhao J., Tang M., Tang H., Wang M., Guan H., Tang L., Zhang H. (2023). Sphingosine 1-phosphate alleviates radiation-induced ferroptosis in ovarian granulosa cells by upregulating glutathione peroxidase 4. Reprod. Toxicol..

[2] Daghestani M.H., Daghestani M.H., Warsy A., El-Ansary A., Omair M.A., Omair M.A., Hassen L.M., Alhumaidhi E.M., Al Qahtani B., Harrath A.H. (2021). Adverse Effects of Selected Markers on the Metabolic and Endocrine Profiles of Obese Women with and Without PCOS. Front. Endocrinol..

[3] Gershon E., Dekel N. (2020). Newly Identified Regulators of Ovarian Folliculogenesis and Ovulation. Int. J. Mol. Sci..

[4] Xie Q., Hong W., Li Y., Ling S., Zhou Z., Dai Y., Wu W., Weng R., Zhong Z., Tan J. (2023). Chitosan oligosaccharide improves ovarian granulosa cells inflammation and oxidative stress in patients with polycystic ovary syndrome. Front. Immunol..

[5] Jozkowiak M., Piotrowska-Kempisty H., Kobylarek D., Gorska N., Mozdziak P., Kempisty B., Rachon D., Spaczynski R.Z. (2022). Endocrine Disrupting Chemicals in Polycystic Ovary Syndrome: The Relevant Role of the Theca and Granulosa Cells in the Pathogenesis of the Ovarian Dysfunction. Cells.

[6] Zhang Q., Ren J., Wang F., Pan M., Cui L., Li M., Qu F. (2022). Mitochondrial and glucose metabolic dysfunctions in granulosa cells induce impaired oocytes of polycystic ovary syndrome through Sirtuin 3. Free Radic. Biol. Med..

[7] Liu Y., Liu H., Li Z., Fan H., Yan X., Liu X., Xuan J., Feng D., Wei X. (2021). The Release of Peripheral Immune Inflammatory Cytokines Promote an Inflammatory Cascade in PCOS Patients via Altering the Follicular Microenvironment. Front. Immunol..

[8] Duffy D.M., Ko C., Jo M., Brannstrom M., Curry T.E. (2019). Ovulation: Parallels with Inflammatory Processes. Endocr. Rev..

[9] Seekford Z.K., Wohlgemuth S.E., Sheldon I.M., Bromfield J.J. (2025). Exposure of bovine granulosa cells to lipopolysaccharide reduces progesterone secretion during luteinization. Biol. Reprod..

[10] Bromfield J.J., Sheldon I.M. (2011). Lipopolysaccharide initiates inflammation in bovine granulosa cells via the TLR4 pathway and perturbs oocyte meiotic progression in vitro. Endocrinology.

[11] Koga A., Thongsiri C., Kudo D., Phuong D.N.D., Iwamoto Y., Fujii W., Nagai-Yoshioka Y., Yamasaki R., Ariyoshi W. (2023). Mechanisms Underlying the Suppression of IL-1β Expression by Magnesium Hydroxide Nanoparticles. Biomedicines.

[12] Fock E.M., Parnova R.G. (2021). Protective Effect of Mitochondria-Targeted Antioxidants against Inflammatory Response to Lipopolysaccharide Challenge: A Review. Pharmaceutics.

[13] Choudhury D., Rong N., Senthil Kumar H.V., Swedick S., Samuel R.Z., Mehrotra P., Toftegaard J., Rajabian N., Thiyagarajan R., Podder A.K. (2024). Proline restores mitochondrial function and reverses aging hallmarks in senescent cells. Cell Rep..

[14] Wang R., Huang S., Wang P., Shi X., Li S., Ye Y., Zhang W., Shi L., Zhou X., Tang X. (2024). Global trends and hotspots in the field of mitochondrial dynamics and hepatocellular carcinoma: A bibliometric analysis from 2007 to 2023. Heliyon.

[15] Lin J., Chang Y., Hu M., Gu Q., Dai J., Nan J., Wang Z., Chen J., Zhong D., Zhou E. (2023). Global trends in research of mitophagy in liver diseases over past two decades: A bibliometric analysis. Heliyon.

[16] Xu S., Dong Y., Chen S., Liu Y., Li Z., Jia X., Briens M., Jiang X., Lin Y., Che L. (2022). 2-Hydroxy-4-Methylselenobutanoic Acid Promotes Follicle Development by Antioxidant Pathway. Front. Nutr..

[17] Mickiewicz B., Villemaire M.L., Sandercock L.E., Jirik F.R., Vogel H.J. (2014). Metabolic changes associated with selenium deficiency in mice. Biometals.

[18] Li Y., Liu J.X., Xiong J.L., Wang Y.M., Zhang W.X., Wang D.M. (2019). Effect of hydroxyselenomethionine on lactation performance, blood profiles, and transfer efficiency in early-lactating dairy cows. J. Dairy Sci..

[19] Chen J., Zhang F., Guan W., Song H., Tian M., Cheng L., Shi K., Song J., Chen F., Zhang S. (2019). Increasing selenium supply for heat-stressed or actively cooled sows improves piglet preweaning survival, colostrum and milk composition, as well as maternal selenium, antioxidant status and immunoglobulin transfer. J. Trace Elem. Med. Biol..

[20] Zhang L., Liu X.R., Liu J.Z., An X.P., Zhou Z.Q., Cao B.Y., Song Y.X. (2018). Supplemented Organic and Inorganic Selenium Affects Milk Performance and Selenium Concentration in Milk and Tissues in the Guanzhong Dairy Goat. Biol. Trace Elem. Res..

[21] Hosnedlova B., Kepinska M., Skalickova S., Fernandez C., Ruttkay-Nedecky B., Malevu T.D., Sochor J., Baron M., Melcova M., Zidkova J. (2017). A Summary of New Findings on the Biological Effects of Selenium in Selected Animal Species-A Critical Review. Int. J. Mol. Sci..

[22] Kamada H. (2017). Effects of selenium-rich yeast supplementation on the plasma progesterone levels of postpartum dairy cows. Asian-Australas. J. Anim. Sci..

[23] Chen Y., Zhao Y.F., Yang J., Jing H.Y., Liang W., Chen M.Y., Yang M., Wang Y., Guo M.Y. (2020). Selenium alleviates lipopolysaccharide-induced endometritis via regulating the recruitment of TLR4 into lipid rafts in mice. Food Funct..

[24] Li H., Wang H., Cui L., Liu K., Guo L., Li J., Dong J. (2024). The effect of selenium on the proliferation of bovine endometrial epithelial cells in a lipopolysaccharide-induced damage model. BMC Vet. Res..

[25] Wang Y., Li Q., Ma Z., Xu H., Peng F., Chen B., Ma B., Qin L., Lan J., Li Y. (2023). β-Nicotinamide Mononucleotide Alleviates Hydrogen Peroxide-Induced Cell Cycle Arrest and Death in Ovarian Granulosa Cells. Int. J. Mol. Sci..

[26] Mazloomi S., Sanoeei Farimani M., Tayebinia H., Karimi J., Amiri I., Abbasi E., Khodadadi I. (2022). The Association of Mitochondrial Translocator Protein and Voltage-Dependent Anion Channel-1 in Granulosa Cells with Estradiol Levels and Presence of Immature Follicles in Polycystic Ovary Syndrome. J. Reprod. Infertil..

[27] Jiang K., Xie C., Li Z., Zeng H., Zhao Y., Shi Z. (2022). Selenium Intake and its Interaction with Iron Intake Are Associated with Cognitive Functions in Chinese Adults: A Longitudinal Study. Nutrients.

[28] Surai P.F., Kochish I.I. (2020). Food for thought: Nano-selenium in poultry nutrition and health. Anim. Health Res. Rev..

[29] Minich W.B. (2022). Selenium Metabolism and Biosynthesis of Selenoproteins in the Human Body. Biochemistry.

[30] Zhao J., Dong L., Lin Z., Sui X., Wang Y., Li L., Liu T., Liu J. (2023). Effects of selenium supplementation on Polycystic Ovarian Syndrome: A systematic review and meta-analysis on randomized clinical trials. BMC Endocr. Disord..

[31] Lee C.S., Hwang G., Nam Y.W., Hwang C.H., Song J. (2023). IKK-mediated TRAF6 and RIPK1 interaction stifles cell death complex assembly leading to the suppression of TNF-α-induced cell death. Cell Death Differ..

[32] Mehta N.N., deGoma E., Shapiro M.D. (2024). IL-6 and Cardiovascular Risk: A Narrative Review. Curr. Atheroscler. Rep..

[33] He F., Liu Y., Li T., Ma Q., Yongmei Z., He P., Xiong C. (2022). MicroRNA-146 attenuates lipopolysaccharide induced ovarian dysfunction by inhibiting the TLR4/NF- κB signaling pathway. Bioengineered.

[34] Mehdi Y., Dufrasne I. (2016). Selenium in Cattle: A Review. Molecules.

[35] Sun J., Liu Q., Zhang X., Dun S., Liu L. (2022). Mitochondrial hijacking: A potential mechanism for SARS-CoV-2 to impair female fertility. Med. Hypotheses.

[36] Su X., Yang D., Hu Y., Yuan Y., Song L. (2023). Berberine suppressed sarcopenia insulin resistance through SIRT1-mediated mitophagy. Open Life Sci..

[37] Yildirim R.M., Seli E. (2024). The role of mitochondrial dynamics in oocyte and early embryo development. Semin. Cell Dev. Biol..

[38] Xia D., Liu Y., Wu P., Wei D. (2023). Current Advances of Mitochondrial Dysfunction and Cardiovascular Disease and Promising Therapeutic Strategies. Am. J. Pathol..

[39] Shimura T., Shiga R., Sasatani M., Kamiya K., Ushiyama A. (2022). Melatonin and MitoEbselen-2 Are Radioprotective Agents to Mitochondria. Genes.

[40] Fernández-Lázaro D., Fernandez-Lazaro C.I., Mielgo-Ayuso J., Navascués L.J., Córdova Martínez A., Seco-Calvo J. (2020). The Role of Selenium Mineral Trace Element in Exercise: Antioxidant Defense System, Muscle Performance, Hormone Response, and Athletic Performance. A Systematic Review. Nutrients.

[41] Wen T., Liu T., Chen H., Liu Q., Shen X., Hu Q. (2024). Demethylzeylasteral alleviates inflammation and colitis via dual suppression of NF-κB and STAT3/5 by targeting IKKα/β and JAK2. Int. Immunopharmacol..

[42] Yan J., Chen L., Zhang L., Zhang Z., Zhao Y., Wang Y., Ou J. (2022). New Insights Into the Persistent Effects of Acute Exposure to AFB_1_ on Rat Liver. Front. Microbiol..

[43] Heller A., Ulstrup J. (2021). Detlev Müller’s Discovery of Glucose Oxidase in 1925. Anal. Chem..

[44] Fan X., Fu H., Xie N., Guo H., Fu T., Shan Y. (2021). Inhibition of JAK2/STAT3 signaling pathway by panaxadiol limits the progression of pancreatic cancer. Aging.

[45] García de la Cadena S., Massieu L. (2016). Caspases and their role in inflammation and ischemic neuronal death. Focus on caspase-12. Apoptosis.

[46] Wu Y.C., Hsu S.P., Hu M.C., Lan Y.T., Yeh E.T.H., Yang F.M. (2022). PEP-sNASP Peptide Alleviates LPS-Induced Acute Lung Injury Through the TLR4/TRAF6 Axis. Front. Med..

[47] Basini G., Tamanini C. (2000). Selenium stimulates estradiol production in bovine granulosa cells: Possible involvement of nitric oxide. Domest. Anim. Endocrinol..

[48] Yao X., Ei-Samahy M.A., Fan L., Zheng L., Jin Y., Pang J., Zhang G., Liu Z., Wang F. (2018). In vitro influence of selenium on the proliferation of and steroidogenesis in goat luteinized granulosa cells. Theriogenology.

[49] Orisaka M., Miyazaki Y., Shirafuji A., Tamamura C., Tsuyoshi H., Tsang B.K., Yoshida Y. (2021). The role of pituitary gonadotropins and intraovarian regulators in follicle development: A mini-review. Reprod. Med. Biol..

[50] Vazakidou P., Evangelista S., Li T., Lecante L.L., Rosenberg K., Koekkoek J., Salumets A., Velthut-Meikas A., Damdimopoulou P., Mazaud-Guittot S. (2024). The profile of steroid hormones in human fetal and adult ovaries. Reprod. Biol. Endocrinol..

[51] Jefferi N.E.S., Shamhari A., Hamid Z.A., Budin S.B., Zulkifly A.M.Z., Roslan F.N., Taib I.S. (2022). Knowledge Gap in Understanding the Steroidogenic Acute Regulatory Protein Regulation in Steroidogenesis Following Exposure to Bisphenol A and Its Analogues. Biomedicines.

[52] Ojo O.O., Bhadauria S., Rath S.K. (2013). Dose-dependent adverse effects of salinomycin on male reproductive organs and fertility in mice. PLoS ONE.

[53] Bonnet A., Cabau C., Bouchez O., Sarry J., Marsaud N., Foissac S., Woloszyn F., Mulsant P., Mandon-Pepin B. (2013). An overview of gene expression dynamics during early ovarian folliculogenesis: Specificity of follicular compartments and bi-directional dialog. BMC Genom..

[54] Wang M., Li Y., Molenaar A., Li Q., Cao Y., Shen Y., Chen P., Yan J., Gao Y., Li J. (2021). Vitamin E and selenium supplementation synergistically alleviate the injury induced by hydrogen peroxide in bovine granulosa cells. Theriogenology.

